# The wolf in sheep’s clothing: Microtomographic aspects of clinically incipient radiation-related caries

**DOI:** 10.4317/medoral.20886

**Published:** 2016-03-06

**Authors:** Karina Morais-Faria, Rodrigo Neves-Silva, Marcio-Ajudarte Lopes, Ana-Carolina-Prado Ribeiro, Gilberto de Castro Jr, Karina-Gondim-Moutinho da Conceição-Vasconcelos, Thais-Bianca Brandão, Alan-Roger Santos-Silva

**Affiliations:** 1 Oral Diagnosis Department, Semiology Area, Piracicaba Dental School, University of Campinas (UNICAMP), Piracicaba, Sao Paulo, Brazil; 2 Dental Oncology Service, Instituto do Câncer do Estado de São Paulo (ICESP), Faculdade de Medicina da Universidade de São Paulo, Sao Paulo, Brazil; 3 Clinical Oncology Department, Instituto do Câncer do Estado de São Paulo (ICESP), Faculdade de Medicina da Universidade de São Paulo, Sao Paulo, Brazil; 4 Radiotherapy Department, Instituto do Câncer do Estado de São Paulo (ICESP), Faculdade de Medicina da Universidade de São Paulo, Sao Paulo, Brazil

## Abstract

**Background:**

Radiation-related caries (RRC) can cause rapid progression, with a high potential for dental destruction affecting mainly cervical and incisal areas. Unlike the injuries that occur in the conventional caries, incipient RRC present in unusual surfaces have difficult diagnosis and classification stages of cavitation.

**Material and Methods:**

Evaluate the radiographic patterns of demineralization of RRC by using micro-CT. Ten teeth with incipient RRC and 10 teeth with incipient conventional caries (control group) matched by anatomic teeth group and caries affected surfaces were evaluated by X-ray microtomography (micro-CT) Skyscan 1174V2 (50Kv, 1.3 megapixel, Kontich, Belgium). Teeth were placed in a standard position for micro-CT (coronal, transaxial and sagittal sections) during images acquisition. Lesions were classified according to the depth of invasion and relationship with enamel, dentin and pulp.

**Results:**

RRC samples presented deeper lesions with higher involvement of enamel and dentin. Control group presented focal and superficial lesions with lower involvement of enamel and dentin.

**Conclusions:**

Incipient RRC present aggressive microtomographic patterns of demineralization when compared to conventional caries, as indicated by deep lesions, regardless of its clinically incipient aspects.

**Key words:**Head and neck cancer, radiotherapy, microtomography, radiation caries.

## Introduction

Approximately 400,000 cases of oral cancer are diagnosed annually worldwide, most of which will be treated by the combination of surgery, radiotherapy and chemotherapy, a therapeutic combination that has been able to improve survival rates for these patients during the last decade. However, radiation-induced oral toxicities, such as xerostomia, oral infections, trismus, radiation-related caries (RRC) and osteonecrosis often affect these patients, eventually causing loss of masticatory function and negatively impacting quality of life (1).

Nearly 25% of oral cancer patients will develop RRC following head and neck radiotherapy (HNRDT) (2). From a clinical perspective, RRC often start on the labial surfaces at the cervical teeth areas and cause diffuse wear of incisal and occlusal surfaces, leading to an increased enamel friability, enamel delimination, eventually generating teeth crown amputation and broad dentition breakdown (3). The present understanding concerning the etiology of RRC is quite controversial; radiation-induced hyposalivation is considered the most significant etiological factor (4,5).

Systematic dental examinations, early diagnosis and prompt treatment (including restorations and dental extractions, if needed) are essential to maintain oral health status and quality of life in post-HNRDT patients (5-7). In this scenario, radiographic examination is still the most recommended adjunct method in the diagnosis of caries; however, there is only scarce information regarding the radiographic features for RRC as well as the radiographic patterns of demineralization in RRC (5).

X-ray microtomography (micro-CT) systems have been available commercially for over two decades. In recent years, micro-CT has been extensively used in dental science as researchers are becoming increasingly familiar with this technique (8-10). Despite of the potential contribution of micro-CT to investigate tooth pathology, to date, there is no study on RRC. Therefore, the current study presents the microtomographic aspects of clinically incipient RRC, aiming to contribute to better understanding of carious development in patients who have undergone HNRDT.

## Material and Methods

- Patients

**Figure 1:**
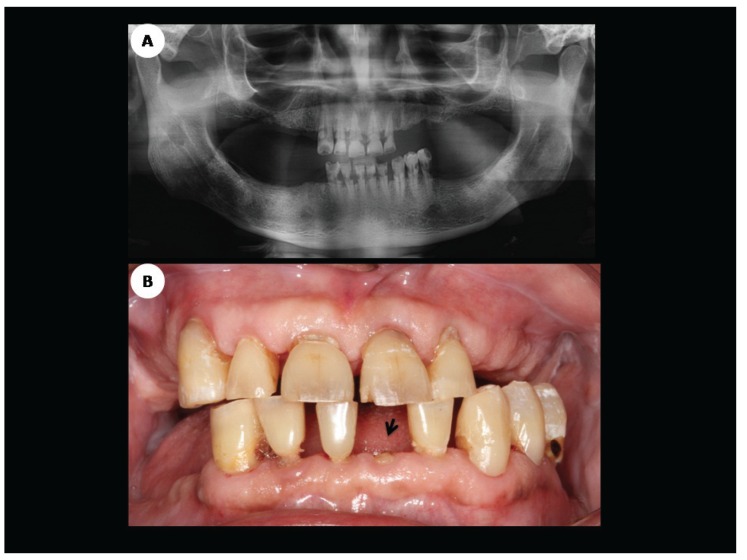
A. Mandible panoramic radiography showing multiple teeth affected by advanced cervical radiation-related caries; B. Clinical aspect of the same patient shown in 1A, demonstrating an apparent incipient case of RRC. Note dental crown amputation of the left mandible central incisor following a few days of the radiographic assessment (arrow).

This retrospective study was approved by the Ethics Committee of the University of Sao Paulo Medical School, Sao Paulo, Brazil (study protocol number 191,171) and all patients enrolled in this study signed the consent form. Ten teeth affected by RRC and extracted due to advanced periodontal disease from six patients who had undergone HNRDT were included in this study. All selected patients were undergoing post-HNRDT dental treatment at the Dental Oncology Service of São Paulo State Cancer Center when the diagnosis of RRC and advanced periodontal disease were clinically established (Fig. 1A,B). Patient´s electronic medical charts were retrieved for epidemiological and pathological characterization and data, such as patient’s gender, age, primary tumor location, among others, were recorded.

All selected patients underwent 3D conformal tridimensional radiotherapy in 6mV linear accelerators on Synergy Platform (Elekta AB, Stockholm, Sweden) with radiation doses ranging from 60 to 70 Gy. Radiotherapy planning was reviewed for all patients for radiation field characterization and total tumor dose estimation on CMS XiO (Elekta CMS Software, St. Louis, Missouri) version 4.60. Selection criteria for the study group samples included teeth affected by RRC that were within the primary radiation ﬁeld during HNRDT. Selection criteria for the control group (n=10) included teeth affected by conventional clinically incipient caries and extracted from healthy patients without relevant medical background (with no history of HNRDT or hypo salivation) due to advanced periodontal disease. Control and RRC samples were matched by patient’s age, anatomic teeth group and caries affected surfaces. All teeth samples studied were fixed in 10% neutral-buffered formalin solution immediately after extraction.

- Sample Evaluation

All teeth were scanned with a micro-CT Skyscan 1174 V2 (50Kv, 1.3 megapixel, Kontich, Belgium) and placed in a standard position for microtomography (coronal, transaxial and sagittal sections) images acquisition. The resulting 2D shadow/transmission images were used to reconstruct the teeth by using a cone beam reconstruction algorithm (NRecon, Version 1.4.2, Skyscan, Kontich, Belgium).

Approximately five hundred tomographic cuts were obtained at each coronal, sagittal and axial plane. After processing the tomographic images, the structures of enamel, dentin and pulp chamber were evaluated, considering depths of caries invasion in a blind manner by a single examiner, following the criteria described by Ekstrand *et al.,* (1997) (11) that was adapted for use in a microtomographic examination. Clinical aspects for severity of tooth damage in RRC and control group samples were macroscopically determined in a blinded study by using a previously validated post-radiation caries index described by Walker *et al.,* (2008) (12). To avoid inter examiner variability, a single examiner performed the study of the depth of caries. All micro-CT images were evaluated once following the same standardized viewing conditions. In order to avoid intraexaminer variability in interpretation of the micro-CT images the investigator performed all assessments in the same viewing room with optimal lighting viewing conditions, no adjustment to display system was allowed.

- Statistical analyses

All the results generated by the micro-CT evaluation were statistically processed by using descriptive statistics, considering absolute values and percentages in the EXCEL™ software.

## Results

The clinicopathologic features of head and neck cancer patients involved in this study are presented in  Table 1.

**Table 1:**
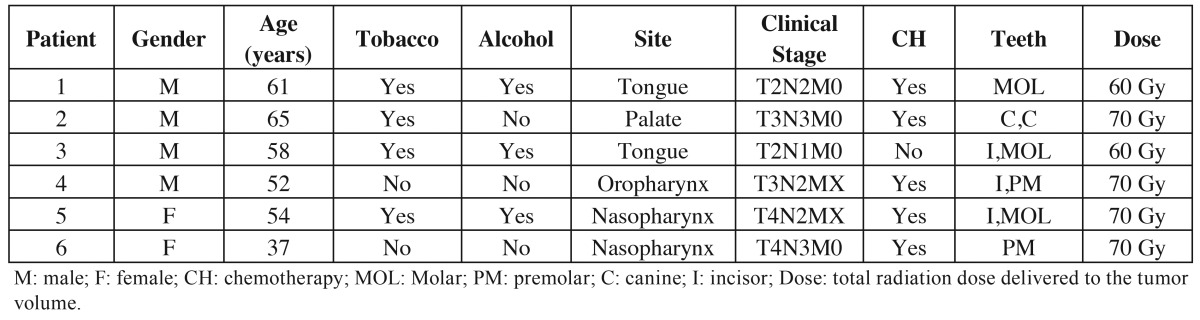
Clinical characteristics of head neck cancer patients.

The results obtained with the macroscopic assessment scores for RRC samples, according to the post-radiation dental index were: 8 (80%) RRC samples received a score of 3 and presented single enamel/tooth structure loss in the focal area (>2mm diameter) or more than 1 focal area of enamel/tooth structure loss; total area of structure loss <1/3 surface area. Two (20%) samples were classified as a score of 2, presenting a single focal area of enamel/tooth structure loss (≤2mm diameter); the surface may also have white line and/or brown stain. For the control group, 8(80%) specimens received a score of 3 and 2 (20%) samples were classified as a score of 2.

**Figure 2:**
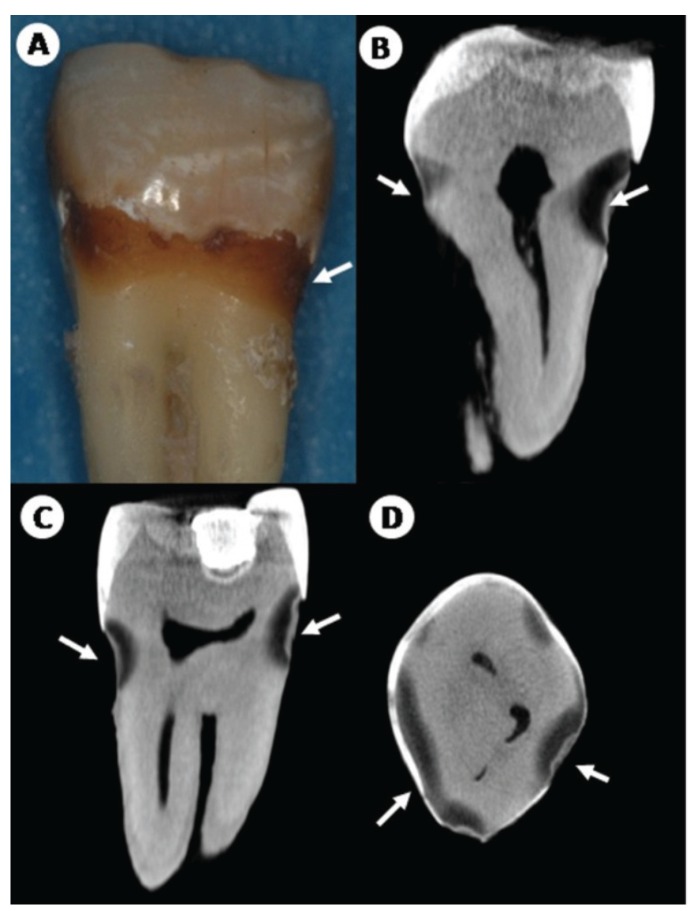
Microtomographic reconstruction images of RRC sample. A. RRC-affected molar presenting macroscopically incipient cervical caries. Coronal (B), sagittal (C) and axial (D) micro-CT slices depictured deep and extensive hipodensities (arrow) affecting enamel, deep dentin and proximity with the pulp chamber.

**Figure 3:**
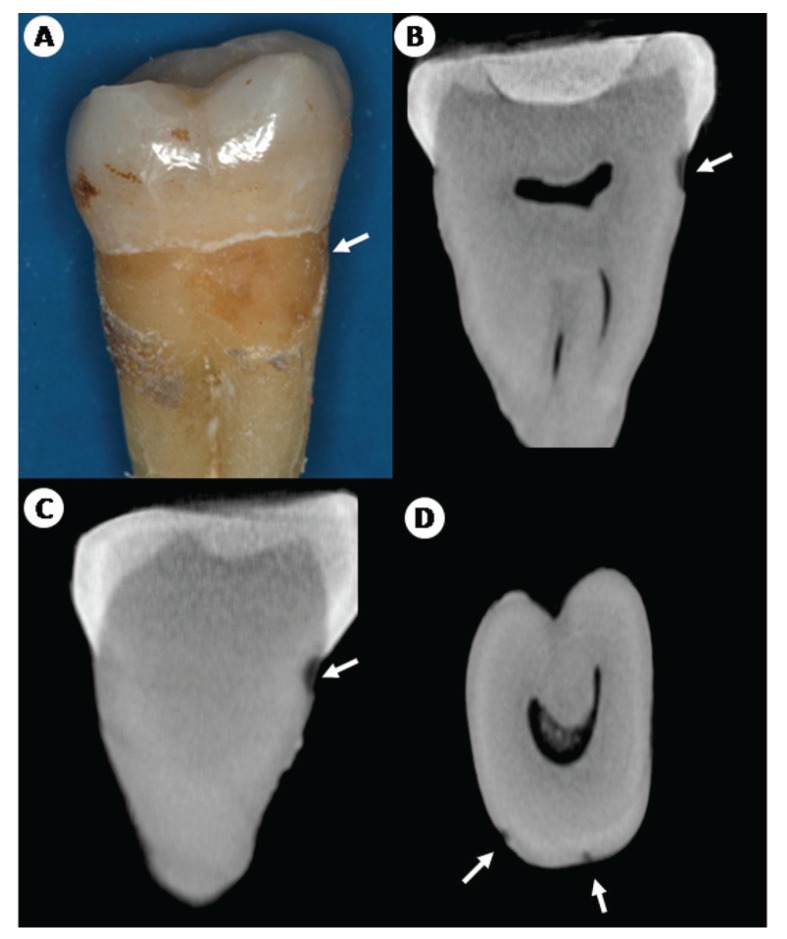
Microtomographic reconstruction images of control sample. A. Control molar specimen with macroscopical cervical incipient caries. Coronal (B), sagittal (C) and axial (D) micro-CT slices presented shallow and discreet hypodense (arrow) images with involvement of superficial enamel, dentin and cervical area.

According to the criteria used for radiographic examination, 8 (80%) RRC samples presented a score of 3 with hypodense images extending to the middle third of the dentine and 2(20%) other RRC samples received a score of 2 and presented hypodense visible images, involving enamel surface through to the dentin but restricted to the outer 1/3 of the dentine teeth (Fig. 2A-D). For the control group, 10 samples (100%) presented a score of 2 with hypodense images visible in the dentine but restricted to the outer third of the dentine (Fig. 3A-D).

## Discussion

Hong *et al.,* (2010) (2) recently performed a systematic review on dental issues in cancer patients and reported that approximately 25% of patients who had undergone HNRDT developed RRC. Despite its high prevalence and relevance to head and neck cancer patient’s quality of life, the pathogenesis of RRC is still unclear and only a few studies focused on a better understanding of the clinical, pathological, radiographic and molecular mechanisms associated with the onset and progression of RRC (1,13,14).

The macroscopic profile of the samples evaluated in the present study is in accordance with previous reports for clinical aspects of incipient RRC (5). Post-radiation samples presented diffuse brown discolorations, affecting non-cavitated enamel surfaces and incisal caries. Apparently, these clinical signals are underestimated in the literature because only a few authors consider them typical manifestations of RRC (5,12). Thereby, we confirm that brown discolorations and incipient RRC represent an important clinical condition that might explain the high potential for destruction of RRC and lead to amputation of dental crowns and complete loss of dentition within short periods of time (5,14).

With respect to the macroscopic analysis, all RRC study samples showed diffuse brown discolorations, affecting enamel smooth surfaces and showing incisal or cervical demineralization areas. Macroscopically, the presence of cavitation was not evident for all RRC samples; on the other hand, it was possible to detect shallow cavitation in all control group samples. Only few previous studies attempted to investigate clinical and radiographic parameters for RRC (5,15,16). Thus, it is very difficult to clinically classify and to standardize the treatment of RRC, which is usually underestimated by patients and clinicians, giving space for RRC lesions to progress and destroy the dentition of post-HNRDT patients.

In addition to the clinical diagnosis that is based on visual inspection of affected surfaces and considers texture and coloration of caries, imaging studies- mainly the radiographic methods-are important techniques to confirm the proper diagnosis of carious lesions, especially in cases of incipient lesions (17,18).

The current study demonstrated that micro-CT was an accurate method to access clinically incipient caries both in RRC and conventional caries.

Only a few authors previously attempted to describe the radiological changes of irradiated teeth. Anneroth *et al.,* (1985) (15) used micro radiographs of ground sections from 54 irradiated teeth and observed RRC on oclusal surfaces and production of secondary dentin. Silva *et al.,* (2009) (5) used periapical radiographs to analyze post-radiation teeth and observed conventional radiolucencies associated with areas affected by cervical and incisal RRC. They also detected radiographic evidence of reactionary dentin indicating that irradiated dentin preserved the ability to react against caries progression. More recently, Hommez *et al.,* (2012) (16) evaluated a total of 36 patients most of whom were affected by RRC after they underwent HNRDT. They used mandible panoramic radiographs and identified a high prevalence of apical periodontitis in teeth affected by RRC. Thus, it becomes evident that little information about the radiographic nature of these brown discolorations and incipient RRC is available and more research is needed. This seems to be the first microtomographic study to assess post-radiation teeth and to observe that in spite of the incipient clinical appearance of RRC, most (80%) RRC samples presented advanced fronts of demineralization characterized by hypodense images extending to the middle third of the dentine; which was not observed in conventional caries (where 100% of the samples presented hypodense images restricted to the outer third of the dentine).

Image study is essential to caries diagnosis; the information is evaluated to decide whether caries is present and what action, if any, should be taken (19,20). The evaluations by imaging methods, such as optical coherence tomography (OCT), are in line with the results presented herein and suggest that nondestructive imaging techniques, such as OCT and micro-CT, may help us to better understand the clinical progression of RRC and could be useful for the improvement of early diagnosis and clinical management of RRC (9).

Dentition breakdown following radiotherapy tends to start within the first year of the conclusion of HNRDT and becomes more severe within time. It is important to bear in mind that post-radiation carious lesions differ considerably in clinical appearance, pattern of development, and progression from conventional caries (1,4,21-24). The results of the current study suggest that RRC often present sub clinical advanced fronts of demineralization, affecting deep dentin and pulp chamber, which may contribute to the above-mentioned aggressive patterns of development and progression.

One of the limitations of the current study was the small sample size. Larger sample sizes would probably lead to more robust results. However, the present study was based on an experimental model of *in vivo* irradiated teeth, which is very difficult to collect because of the risk of post-extraction osteorradionecrosis. Therefore, post-HNRDT tooth extractions should be mainly indicated when there is extensive carious lesion with pulp involvement or advanced periodontal disease and tooth mobility associated with bone loss. Thus, teeth samples affected by incipient RRC are even more rarely extracted and to the best of the authors’ knowledge this is the first study based exclusively on incipient RRC.

When taken together, the presented results may suggest that clinically superficial RRC lesions (especially those characterized by diffuse brown discolorations affecting non-cavitated smooth surface enamel) do not correspond to its tomographic patterns of demineralization, since, from a microtomographic point of view, clinically incipient RRC present advanced lesions showing deep involvement of the dentin and close relationship to the pulp chamber. This radiographic phenomenon could not be observed in conventional clinically incipient caries, where only well-delimited hypodense tomographic images involving superficial enamel and dentin could be seen. For the control group, the microtomographic patterns of demineralization were in accordance with the clinical (macroscopic) carious appearance.

In other words, clinically incipient RRC lesions, such as brown discoloration of enamel and dentin as well as incisal caries should be regarded as “wolves in sheep’s clothing.” In fact, such lesions should be faced as deep carious lesions affecting the dentin and the pulp. More importantly, this study highlights the need for a better understanding of the rapid onset and progression of RRC and urges development of therapeutic protocols for clinically incipient RRC.

In conclusion, micro-CT was able to originally demonstrate that the radiographic patterns of demineralization for RRC are significantly more aggressive than its clinical incipient appearance suggests.
